# Beyond CCR7: dendritic cell migration in type 2 inflammation

**DOI:** 10.3389/fimmu.2025.1558228

**Published:** 2025-02-28

**Authors:** Audrey Meloun, Beatriz León

**Affiliations:** ^1^ Innate Cells and Th2 Immunity Section, Laboratory of Allergic Diseases, National Institute of Allergy and Infectious Diseases, National Institutes of Health, Bethesda, MD, United States; ^2^ Department of Microbiology, University of Alabama at Birmingham, Birmingham, AL, United States

**Keywords:** dendritic cell migration, type 2 immune responses, CCR7, cysteinyl leukotrienes (cysLTs), sphingosine-1-phosphate (S1P), 7α,25-Dihydroxycholesterol (7α,25-OHC), prostaglandins (PGs), EBI2 (GPR183)

## Abstract

Conventional dendritic cells (cDCs) are crucial antigen-presenting cells that initiate and regulate T cell responses, thereby shaping immunity against pathogens, innocuous antigens, tumors, and self-antigens. The migration of cDCs from peripheral tissues to draining lymph nodes (dLNs) is essential for their function in immune surveillance. This migration allows cDCs to convey the conditions of peripheral tissues to antigen-specific T cells in the dLNs, facilitating effective immune responses. Migration is primarily mediated by chemokine receptor CCR7, which is upregulated in response to homeostatic and inflammatory cues, guiding cDCs to dLNs. However, during type 2 immune responses, such as those triggered by parasites or allergens, a paradox arises—cDCs exhibit robust migration to dLNs despite low CCR7 expression. This review discusses how type 2 inflammation relies on additional signaling pathways, including those induced by membrane-derived bioactive lipid mediators like eicosanoids, sphingolipids, and oxysterols, which cooperate with CCR7 to enhance cDC migration and T helper 2 (Th2) differentiation. We explore the potential regulatory mechanisms of cDC migration in type 2 immunity, offering insights into the differential control of cDC trafficking in diverse immune contexts and its impact on immune responses.

## Introduction

Conventional dendritic cells (cDCs) are professional antigen-presenting cells that play a critical role in initiating and regulating T cell responses (1). Acting as immune sentinels, cDCs patrol peripheral tissues, continuously monitoring for external threats, tissue damage, or homeostatic signals. Under these conditions, they capture antigens, process them, and migrate to regional lymphoid organs and tissues, where they present the antigens to naïve T cells. Through this process, cDCs initiate and shape T cell responses, promoting either immunity against pathogens and tumors or tolerance to innocuous and self-antigens. Depending on the context, they can drive the differentiation of CD4^+^ T cells into various cytokine-skewed subsets (such as T helper 1 (Th1), Th2, Th17, or regulatory T cells (Tregs) and promote their functional diversification into T follicular helper (Tfh) cells, as well as effector and memory cells (2–4). Additionally, they initiate and support CD8^+^ T cell responses (5, 6).

cDCs are broadly classified into two main subsets: cDC1 and cDC2, which are derived from distinct, committed bone marrow precursors (7–9). Both subsets uptake antigens, process them, and promote specific adaptive immune responses based on the nature of the antigen and the surrounding inflammatory environment (1, 3). cDC1s specialize in cross-presenting antigens to CD8^+^ T cells, which is critical for antiviral and antitumor immunity, also known as type 1 immunity (10). cDC2s are a heterogeneous population primarily known for priming CD4^+^ T cells to facilitate adaptive immune responses against extracellular pathogens, including Th17 and Th2 cell responses (2, 3, 11–13). Additionally, cDC2s can support specific aspects of type 1 immunity (5, 6, 14). Recent studies have identified additional subdivisions within cDC2s, specifically in the non-migratory resident population of the spleen, referred to as cDC2A and cDC2B (15–17). However, it remains debated whether these cDC2 subsets are consistent across different tissues (18) or if they represent ontogenically distinct subsets with pre-established functions (15–17). Additionally, environmental stimuli shape the transcriptional programs of cDC2s, driving their functional heterogeneity and critically contributing to their diversification across tissues and under various conditions, including homeostasis, infection, and inflammation (19). Thus, the current debate centers on whether the functional heterogeneity of cDC2s is dictated by ontogenically distinct subsets, such as cDC2A and cDC2B, or by environmental stimuli encountered in the tissue during differentiation from precursors (19). Recent evidence has also identified a distinct DC3 subset; however, some controversy exists regarding whether they should be classified as cDCs or instead belong to a different lineage of antigen-presenting cells (17, 20, 21). If classified outside the cDC lineage, DC3s could be grouped with other DC subsets, including plasmacytoid DCs (pDCs) and monocyte-derived DCs (moDCs), which primarily function as innate cytokine producers to promote antiviral responses or tissue inflammation, rather than antigen presentation for initiating T cell responses (22–24).

The ability of cDCs to migrate from barrier tissues to lymphoid tissues underpins their role in immunity. DCs continuously survey peripheral tissues, capturing antigens and transporting them to draining lymph nodes (dLNs) to facilitate encounters with antigen-specific T cells, thereby initiating T cell priming (25, 26). This migration is essential not only for effective immune surveillance and the timely activation of adaptive immune responses when needed but also for maintaining T cell tolerance to prevent inappropriate immune reactions (26, 27). cDC migration from peripheral tissues to dLNs occurs through the lymphatics. This process is mediated by the upregulation of the chemokine receptor CCR7 on cDCs, which facilitates their migration in response to the chemokine ligands CCL21 and CCL19 (25, 26). These chemokines are expressed by lymphatic endothelial cells and stromal cells within the LNs, guiding cDCs through the lymphatic vessels towards the dLNs to ensure efficient antigen delivery and interaction with T cells (28–30). Under homeostatic conditions, tissue cDCs upregulate CCR7 following the activation of cell shape-sensing pathways, triggered by the physical constraints encountered during continuous patrolling of peripheral tissues (31). Additionally, efferocytosis of apoptotic cells and the activation of cholesterol metabolism pathways can similarly induce CCR7 expression (32, 33). These mechanisms promote steady-state, low-paced migration to dLNs, ensuring the presentation of self-antigens and contributing to the maintenance of T cell tolerance (31, 33). During invasion by viruses, bacteria, and fungi, a more robust upregulation of CCR7 occurs in cDCs. This upregulation is triggered by microbial sensing through pattern recognition receptors (PRRs) or by pro-inflammatory cytokine signaling indirectly triggered by these pathogens (34). Additionally, this activation induces the stabilization of HIF-1α, which leads to a switch to the HIF-1α-dependent transcriptional pathway in DCs, resulting in metabolic reprogramming toward glycolysis (35, 36). The increased expression of CCR7, combined with glycolytic metabolism, enhances the migration of cDCs to dLNs (35, 37). This enhanced migration, coupled with a transcriptional profile optimized for antigen presentation and cytokine production, enables effective T cell activation and subsequent Th1 and Th17 differentiation, facilitating a tailored immune response to combat the invasive pathogens (26). During exposure to stimuli such as parasites and allergens that induce Th2 cell differentiation and type 2 immunity, cDCs exhibit robust migration to the dLNs, similar to the migration observed with Th1/Th17-inducing pathogens (13, 38, 39). However, this migration occurs despite low CCR7 expression on cDCs, as Th2-inducing exposures do not upregulate CCR7 beyond basal levels and may, in fact, downregulate it (25, 39, 40). This presents a contradiction regarding whether cDCs can enhance migration to the dLNs while maintaining low CCR7 activity. In this review, we discuss evidence suggesting that cDC migration during type 2 inflammation relies on additional signaling mechanisms that work alongside CCR7 to facilitate cDC migration to the dLNs and ensure effective Th2 response activation. We will particularly focus on the role of membrane-derived bioactive lipid mediators, including arachidonic acid (AA)-derived eicosanoids [e.g., prostaglandins and leukotrienes), sphingolipids (such as sphingosine-1-phosphate (S1P)], and cholesterol-derived oxysterols. These mediators, released during homeostasis and inflammatory conditions, play key roles in supporting CCR7-dependent cDC migration. This review emphasizes these complementary mechanisms that enhance CCR7-mediated cDC migration, with a particular focus on their role in type 2 immunity. Additionally, we will explore whether low CCR7 expression is crucial for the effective induction of Th2 responses, shedding light on the differential regulation of cDC migration in various immune contexts.

## CCR7-dependent migration of cDCs from tissue to dLNs: ligands and key steps

CCR7 is critically important for guiding cDC migration from tissues to the dLNs (22, 41–43). CCR7 is a G protein-coupled receptor activated by the chemokines CCL21 and CCL19. When these chemokines bind to CCR7 on cDCs, they cause a conformational change in the receptor, activating associated G-proteins, primarily Gαi. This activation facilitates the exchange of GDP for GTP on the Gα subunit and the dissociation of the Gβγ subunits, triggering the release of intracellular calcium and initiating the MAPK (ERK1/2), PI3K/Akt, and Rho/Pyk2/cofilin signaling pathways. These pathways drive cytoskeletal rearrangements, cell polarization, and enhanced migration, facilitating the directed movement (chemotaxis) of cDCs toward chemokine gradients of CCL21 and CCL19 (22, 37, 44, 45). CCL19 and CCL21 have similar binding affinities for CCR7 (46). In 3D environments, DCs show comparable sensitivities to both chemokines at lower gradients. However, DCs migrate more efficiently toward higher gradients of CCL21, indicating that CCL21 is a more potent directional cue for DCs than CCL19 (42, 47). Additionally, CCL19 and CCL21 differentially influence receptor internalization and recycling. CCL19 induces stronger β-arrestin activation and clathrin-dependent desensitization, resulting in more rapid internalization and recycling of CCR7 compared to CCL21 (48, 49). CCL19-induced receptor recycling not only plays a role in transient desensitization to CCL19 signaling—allowing DCs to reset their sensitivity to new chemokine gradients—but also helps shape local CCL19 gradients. By internalizing CCL19, CCR7 depletes it from the surrounding environment, enabling DCs to create their own chemokine gradient. This process facilitates chemotaxis even in uniformly distributed CCL19 fields. Moreover, it guides other CCR7-expressing immune cells, particularly T cells, aiding their co-encounter (50). Overall, while CCL21 provides a stronger directional cue and drives more efficient chemotactic responses in DCs under gradient conditions, CCL19 enables DC chemotaxis in uniform concentrations and co-guides other CCR7-expressing cells. Therefore, CCL21-driven chemotaxis may be vital for guiding DCs directionally toward the dLNs, whereas the ability of DCs to generate CCL19 gradients may be crucial for guiding co-migrating T cells within the dLNs and facilitating DC-T cell interactions.

**Figure 1:**
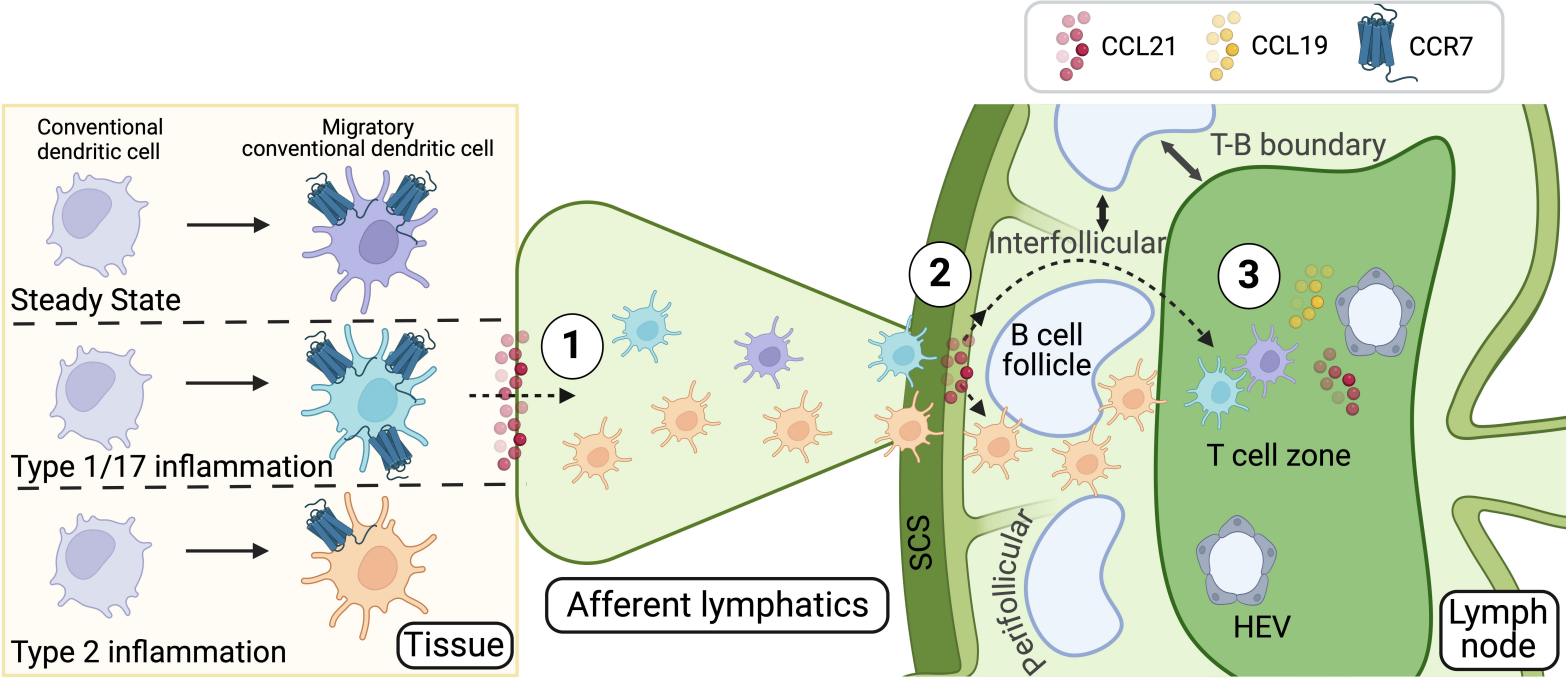
CCR7-dependent steps in cDC migration and localization in the dLN. CCR7 and its ligands, CCL21 and CCL19, orchestrate the migration and positioning of cDCs during both steady-state and immune responses. cDC migration from peripheral tissues to the dLN occurs in three distinct steps, each with specific roles for CCL21 and CCL19: 1) Entry into afferent lymphatics: tissue-resident cDCs enter lymphatic capillaries guided by CCL21, which is constitutively expressed by lymphatic endothelial cells. 2) Transition through the SCS: after traveling passively through afferent lymphatic vessels, cDCs reach the SCS of the dLN, where ACKR4 shapes CCL21 gradients, enabling cDCs to cross into the dLN parenchyma. 3) Positioning in the T Cell Areas: within the dLN, cDCs follow CCL21 and CCL19 gradients to localize in T cell areas, inducing tolerance to self-antigens or facilitating pathogen-specific responses, such as Th1 and Th17 immunity. In type 2 immunity, characterized by low CCR7 expression on cDCs, their migration to the dLN remains efficient, but their localization is restricted to perifollicular areas, where they support Th2 responses. These CCR7-regulated positioning differences underscore the role of cDCs in tailoring immune responses based on the pathogen type, inflammatory context, or steady-state conditions. The mechanisms enabling cDC trafficking during type 2 inflammation, despite low CCR7 activity, remain an open question. Figure created with Biorender.

The seemingly distinct roles of the two CCR7 ligands, CCL21 and CCL19, are likely especially important because they are produced in different locations, enabling distinct and coordinated actions along the pathway from the tissue to the dLN. Indeed, CCR7 regulates the migration of cDCs from peripheral tissues to dLNs through three principal steps, with each ligand playing a key role at specific stages (25) (**Figure 1**). First, tissue cDCs must migrate toward and enter the lymphatic system through nearby lymphatic capillaries. This process is guided by the ligand CCL21, which is constitutively expressed by lymphatic endothelial cells (25, 42, 51). Once inside the lymphatics, cDCs passively travel through larger afferent lymphatic vessels toward the dLN and are delivered into the subcapsular sinus (SCS) of the dLN. In the SCS, cDCs follow CCL21 gradients shaped by the atypical chemokine scavenger receptor ACKR4 on the outer wall (ceiling) of the SCS, enabling them to cross the inner wall (floor) of the SCS and enter the dLN parenchyma (52). The third step involves CCR7 guiding the migration of cDCs into the T cell area of the dLN by following CCL21 and CCL19 gradients (28, 53). This precise navigation is particularly important in conditions where cDCs strongly express CCR7, allowing them to efficiently migrate to the dLN and home within the T cell areas. Upon encountering pathogens like viruses, bacteria, or fungi, cDCs exhibit robust CCR7 expression, facilitating their positioning within T cell areas and promoting the induction of Th1 and Th17 responses (25, 54). This underscores the importance of proper cDC positioning within the T cell areas of dLNs for effectively initiating these types of immune responses. In contrast, during type 2 immunity—such as responses to parasites or allergens—cDCs exhibit low CCR7 expression (25, 39, 40). Although they still migrate in significant numbers to the dLN, they preferentially localize near SCS, perifollicular areas, and T-B cell borders (4, 39, 55) (**Figure 1**). Low CCR7 activity prevents them from efficiently crossing the inner wall of the SCS and penetrating deep into the T cell zones, instead remaining around regions surrounding B cell follicles (39). This perifollicular localization of cDCs and their interactions with specific T cells in these areas are essential for the induction of Th2 responses (39). Therefore, low CCR7 activity during type 2 immunity ensures the correct positioning of cDCs within the dLN, facilitating effective T cell interactions in an environment that promotes Th2 cell development (11, 25). Overall, these differences in cDC localization underscore how cDCs orchestrate distinct T cell responses based on the nature of the pathogen, the inflammatory environment, and their specific positioning within the LN. This precise localization is critically regulated by CCR7 expression levels in cDCs. Given the dual role of CCR7 in guiding cDCs to the dLN and positioning them within the deep T cell zones, an important question arises: how do cDCs, which inherently exhibit low CCR7 activity during type 2 inflammation to avoid localization in T cell zones, still efficiently enter peripheral lymphatic capillaries and reach the dLN in high numbers? It is intriguing to consider whether additional signals, beyond CCR7, support cDC trafficking toward the LN and their localization, particularly during type 2 immune responses.

## The role of eicosanoids in regulating cDC migration

**Figure 2:**
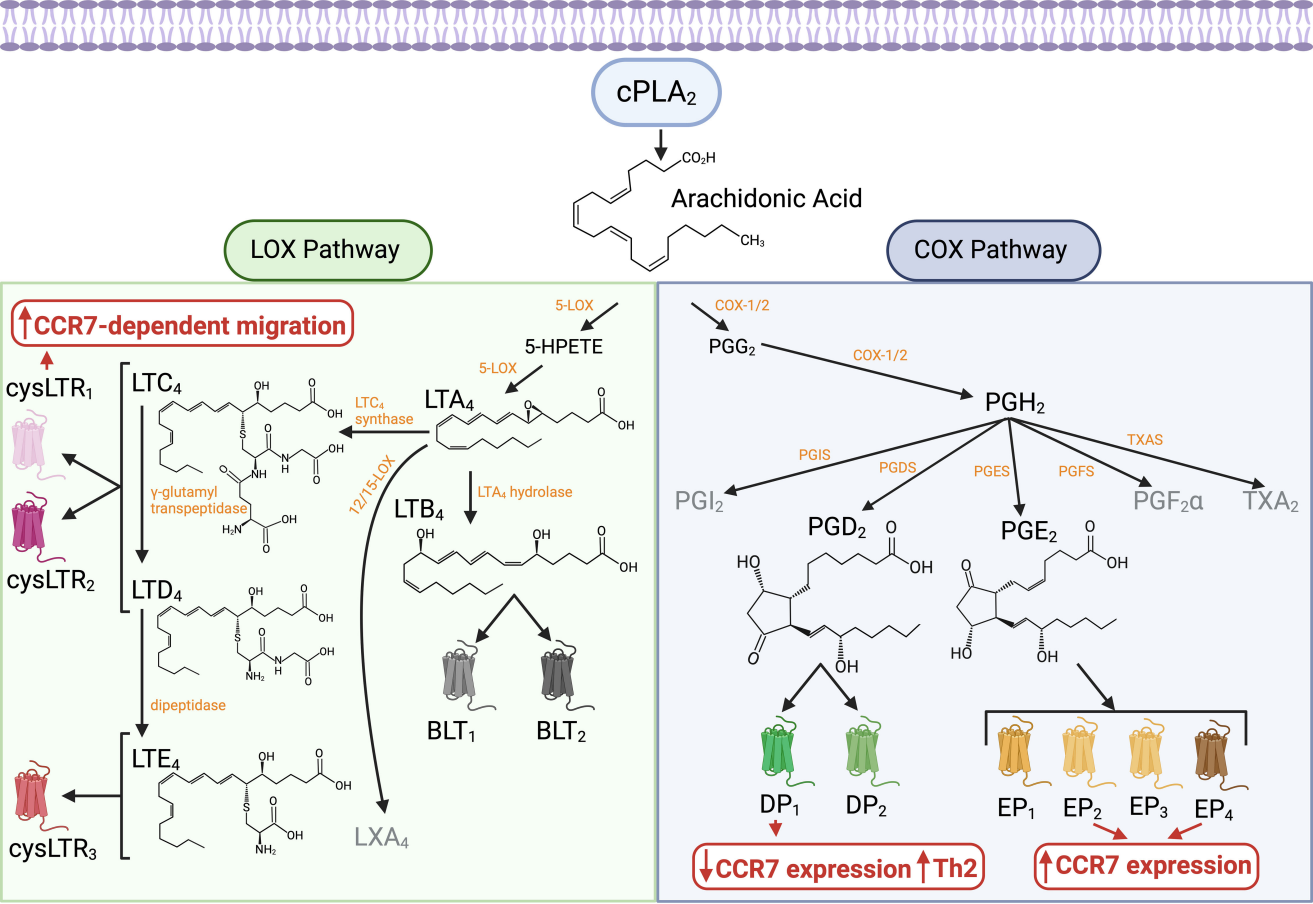
Biosynthesis pathways and receptors of AA-derived eicosanoids and their influence on cDC migration. Eicosanoids are bioactive lipids primarily derived from AA, a 20-carbon polyunsaturated fatty acid released from membrane phospholipids by cPLA2. AA metabolism occurs through two main biosynthetic pathways: the COX pathway and the LOX pathway. In the COX pathway, constitutive COX-1 and inducible COX-2 enzymes convert AA into PGH2, a precursor for various prostanoids, including prostaglandins, prostacyclin, and thromboxanes. PGE2, synthesized via COX-2 and PGES, acts on EP1-4 receptors. PGE2 signaling through EP2 and EP4 receptors on cDCs enhances CCR7 expression and function, promoting cDC migration toward dLNs and facilitating their localization within the T cell areas. PGD2, produced through the COX-2/PGDS pathway, signals via DP1 and DP2 (CRTH2) receptors. Unlike PGE2, PGD2 signaling through DP1 receptors on DCs downregulates CCR7 expression and enhances their ability to promote Th2 cell differentiation. In the LOX pathway, 5-LOX converts AA into 5-HPETE and LTA4. LTA4 is further processed into leukotrienes. LTC4 synthase/LTC4S catalyzes the formation of LTC4, which is subsequently converted to LTD4 and LTE4. LTC4, LTD4, and LTE4, collectively known as cysteinyl leukotrienes, interact with CysLT1, CysLT2, and CysLT3 receptors. On DCs, CysLT1 receptor signaling supports CCR7-mediated mobilization of cDCs from peripheral tissues to dLNs. Additionally, LTA4 hydrolase/LTA4H produces LTB4, which binds to BLT1 and BLT2 receptors and acts as a potent chemoattractant for neutrophils. Overall, during steady-state and type 1/type 17 immune responses, the PGE2 signaling pathway enhances CCR7 expression and CCR7-dependent cDC migration. However, during the initiation of type 2 immunity, the LOX and COX pathways are activated and interact, particularly through mediators such as LTC4, LTD4, and PGD2. This coordinated mechanism promotes Th2 cell-mediated immunity while ensuring efficient guidance of DCs to dLNs under conditions of reduced CCR7 activity. Figure created with Biorender.

Eicosanoids are well-characterized bioactive lipids derived primarily from AA, a 20-carbon polyunsaturated fatty acid that is released from membrane phospholipids by cytosolic phospholipase A2 (cPLA2). AA is metabolized through two main biosynthetic pathways (**Figure 2**). In the cyclooxygenase (COX) pathway, constitutive cyclooxygenase-1 (COX-1) and inducible cyclooxygenase-2 (COX-2) convert AA into prostaglandin H2 (PGH2), a precursor for various prostanoids, including prostaglandins, prostacyclin, and thromboxanes (56, 57). In the lipoxygenase (LOX) pathway, lipoxygenase enzymes such as 5-LOX, 12-LOX, and 15-LOX convert AA into hydroperoxyeicosatetraenoic acids (HPETEs) (57). These intermediates are further processed into leukotrienes, which mediate inflammatory and allergic responses, and lipoxins, which exhibit anti-inflammatory and pro-resolving properties (56, 57). Eicosanoids function in both paracrine and autocrine manners by interacting with G protein-coupled receptors on the surface of target cells.

Prostaglandin E2 (PGE2) plays a pivotal role in regulating basal CCR7 expression on cDCs during homeostasis, supporting their steady-state migration to dLNs (31, 32, 58, 59). This migration ensures the presentation of self-antigens, contributing to immune tolerance through the induction and maintenance of Tregs (26, 27). Under homeostatic conditions, PGE2 is produced downstream of cPLA2, which is activated by shape-sensing mechanisms in tissue-patrolling cDCs (31). Activated cPLA2, recruited to the nucleus, utilizes phospholipids from the nuclear membrane to generate AA. AA is subsequently converted into PGE2 via COX-2/PTGS2 and prostaglandin E synthase (PGES) (**Figure 2**), both of which are upregulated by the same shape-sensing mechanisms. PGE2 induces CCR7 expression by signaling through the EP2 and EP4 receptors (58, 60), activating IKKβ and NF-κB in a paracrine manner to drive CCR7 transcription (27, 31). Additionally, efferocytosis of apoptotic cells can enhance PGE2 production, further contributing to CCR7 expression on steady-state DCs, although the precise mechanism remains unclear (32). Overall, basal CCR7 upregulation on tissue cDCs during homeostasis is regulated by autocrine/paracrine PGE2 production, ensuring baseline migration to dLNs and maintaining tolerance to self-antigens. At sites of inflammation, PGE2 production is further amplified by the enzymatic activity of COX-2 and PGES, both of which are highly induced by pathogen-associated molecular patterns (PAMPs), such as bacterial lipopolysaccharide (LPS), and proinflammatory stimuli, including TNF-α, IL-1β, and IL-6 (57, 61). Thus, upon encountering pathogens or the resulting inflammatory milieu, increased production of PGE2 likely contributes to the upregulation of CCR7 expression in cDCs above basal levels. Additionally, PGE2 can increase CCR7-dependent signaling, promoting CCR7 oligomerization (62) and activation of signal transduction pathways (63). This PGE2-dependent enhancement in CCR7 expression and function is likely to amplify cDC migration toward dLNs, contributing to the robust induction of specific T cell responses, particularly Th1 or Th17, against the triggering pathogen products or associated cytokines.

During type 2 inflammation triggered by parasites or allergens, the COX-2 pathway is also induced, leading to the production of various prostaglandins. Among these, prostaglandin D2 (PGD2) is particularly associated with type 2 responses and plays a pivotal role in its pathogenesis (64–67). In contrast, PGE2 is linked to a protective effect against type 2 inflammation (68–70). PGD2 is produced primarily by mast cells through the action of prostaglandin D synthase (PGDS) (65, 71, 72) (**Figure 2**). The production of PGD2 is induced following the activation of mast cells via IgE-dependent or IgE-independent mechanisms (73–75). PGD2 acts on specific receptors, such as DP1 and DP2 (also known as CRTH2), and exhibits chemotactic properties, promoting the recruitment and activation of Th2 cells, type 2 innate lymphoid cells (ILC2s), eosinophils, and basophils (75–79), which are key players in allergic inflammation. IgE-independent mechanisms of mast cell activation through the mast cell receptor MrgprB2/MRGPRX2, mediated by substance P produced by nociceptive sensory neurons after allergen stimulation, have been shown to significantly contribute to Th2 polarization and the initiation of type 2 inflammation (12, 80). This likely occurs by facilitating DC migration to the dLNs in a way that favors the development of Th2 cell responses (12, 81). Mast cell-derived PGD2 may contribute to this process by modulating DC migration and function through signaling via the DP1 receptor. PGD2 instructs DCs to polarize CD4^+^ T cells toward Th2 responses, and this effect is dependent on the expression of DP1 on DCs (82, 83). One of the mechanisms is through the suppression of Th1/Th17 polarizing cytokines such as IL-12 or IL-6, via inhibition of NF-κB signaling (82–85). The lack of cytokine secretion by cDCs allows them to support Th2 differentiation (2, 11, 86). Secondly, PGD2 prevents the upregulation of CCR7 in DCs, counteracting this effect induced by exposure to PAMPs (e.g., LPS) or inflammatory cytokines such as TNF-α (82, 85, 87). Low CCR7 activity induced by PGD2 hinders DC migration toward CCL19, thereby preventing their homing into the T cell areas of the dLN—a hallmark of the cDCs that induce Th2 responses (25, 39). However, this reduced CCR7 expression in cDCs impairs efficient migration and the stimulation of T cell responses in other inflammatory contexts, such as TNF-α-induced activation (88) or viral infection (89). Overall, these findings demonstrate that PGD2 influences DC migration and function, specifically promoting Th2 cell-based immunity while suppressing other T helper cell responses.

Besides the COX-2-PGDS-PGD2 pathway, the LOX pathway is another eicosanoid-related pathway that is highly induced during type 2 inflammation, producing leukotrienes that contribute to pathogenesis in the context of allergy and asthma. Cysteinyl leukotrienes—specifically LTC4, LTD4, and LTE4—are key mediators synthesized through the action of the 5-LOX enzyme (56, 90). 5-LOX converts AA into leukotriene A4 (LTA4), which is subsequently converted into LTC4 by leukotriene C4 synthase (LTC4S) and exported extracellularly via MRP family transporters. Once released into the extracellular space, LTC4 is converted to LTD4 by gamma-glutamyl transpeptidase (GGT) and subsequently to LTE4 by dipeptidase (56). Cysteinyl leukotrienes signal through G protein-coupled receptors—CysLT1, CysLT2, and CysLT3 (also known as GPR99 or OXGR1)—to mediate their actions (**Figure 2**). CysLT1 and CysLT2 receptors exhibit overlapping affinities for LTC4 and LTD4, whereas CysLT3 preferentially binds LTE4. Additionally, leukotriene B4 (LTB4) is produced by 5-LOX and then converted by leukotriene A4 hydrolase (LTA4H) (56). LTB4 signals through BLT1 and BLT2 receptors (**Figure 2**) and acts as a potent chemoattractant for neutrophils (91–93). It has been proposed to play a role in neutrophilic asthma (also referred to as type 2-low asthma), which is driven by Th1/Th17-mediated inflammation and neutrophil recruitment (94, 95). This contrasts with the pro-type 2 inflammatory role of cysteinyl leukotrienes (96). Moreover, COX-dependent PGE2 suppresses 5-LOX activity (97, 98) and inhibits the CysLT1 receptor (99), thereby reducing the capacity of cells to produce or respond to cysteinyl leukotrienes. These findings highlight cross-regulation among eicosanoid-related pathways and emphasize the predominance of specific pathways in modulating distinct immune responses.

Cysteinyl leukotrienes are primarily produced by mast cells and basophils through IgE-dependent mechanisms (96). Additionally, these cells can generate cysteinyl leukotrienes in response to interleukin-33 (IL-33) stimulation (100). Other innate cells, such as DCs (101), and specialized epithelial tuft cells (102, 103), can contribute to leukotriene production after activation by allergens, helminths, or extracellular ATP. Cysteinyl leukotrienes contribute to various aspects of type 2 allergic inflammation by promoting bronchoconstriction, mucus production, increased vascular permeability, and the recruitment of eosinophils and basophils, as well as the activation of ILC2 and Th2 cells (96, 104–108). Moreover, the LOX pathway interacts with other inflammatory pathways, such as the COX pathway, to amplify the inflammatory response. For instance, the cross-talk between cysteinyl leukotrienes and PGD2 enhances the recruitment and activation of Th2 cells, eosinophils, and mast cells, further driving type 2 inflammation (104, 109–111). The use of 5-LOX inhibitors or leukotriene receptor antagonists, such as the CysLT1 antagonists montelukast, zafirlukast, and pranlukast, has been shown to reduce asthma symptoms and improve lung function, highlighting the significant role of this pathway in the pathogenesis of type 2 driven diseases (112, 113). Overall, the induction of the LOX pathway during type 2 inflammation and its contribution to allergy and asthma pathogenesis underscore the importance of this pathway in driving immune mechanisms associated with type 2 immunity.

Cysteinyl leukotrienes not only amplify type 2 responses but also play a critical role in the initiation of allergic airway inflammation. Cysteinyl leukotrienes achieve this effect by modulating DC functions to promote Th2 cell priming (101). This outcome is mediated through signaling via the CysLT1 receptor on DCs (114, 115). One proposed mechanism involves the regulation of DC migration. *In vitro*, LTD4 induces DC chemotaxis and enhances their migration in response to CCR7 ligands (99). *In vivo* mouse models demonstrate that cysteinyl leukotrienes, including LTC4 and LTD4, are essential for mobilizing DCs from the skin to dLNs, facilitating CCR7-dependent migration (116). Similarly, in the lungs, cysteinyl leukotrienes regulate the migration of airway CCR7-expressing cells to dLNs (117). These findings suggest that cysteinyl leukotrienes assist CCR7 in mobilizing DCs from peripheral tissues to dLNs during the initiation of type 2 responses (**Figure 2**). Although CCR7 expression is essential for DC migration, it alone may not suffice to ensure efficient mobilization during the initiation of type 2 responses, particularly since CCR7 expression remains low in DCs during type 2 inflammation. Therefore, cooperative signaling with cysteinyl leukotrienes may play a complementary role in facilitating this process. This effect seems to depend on the CysLT1 receptor on DCs, whereas CysLT2 appears to exert negative regulatory signals, potentially inhibiting CysLT1 activity (99, 118). In conclusion, during the initiation of type 2 immunity, the LOX and COX pathways are activated and seem to interact, particularly through mediators such as LTC4, LTD4, and PGD2, to influence DC migration and function. This interplay promotes Th2 cell-mediated immunity while ensuring efficient guidance of DCs to dLNs, even under conditions of low CCR7 activity.

## The role of sphingolipids in regulating cDC migration

**Figure 3:**
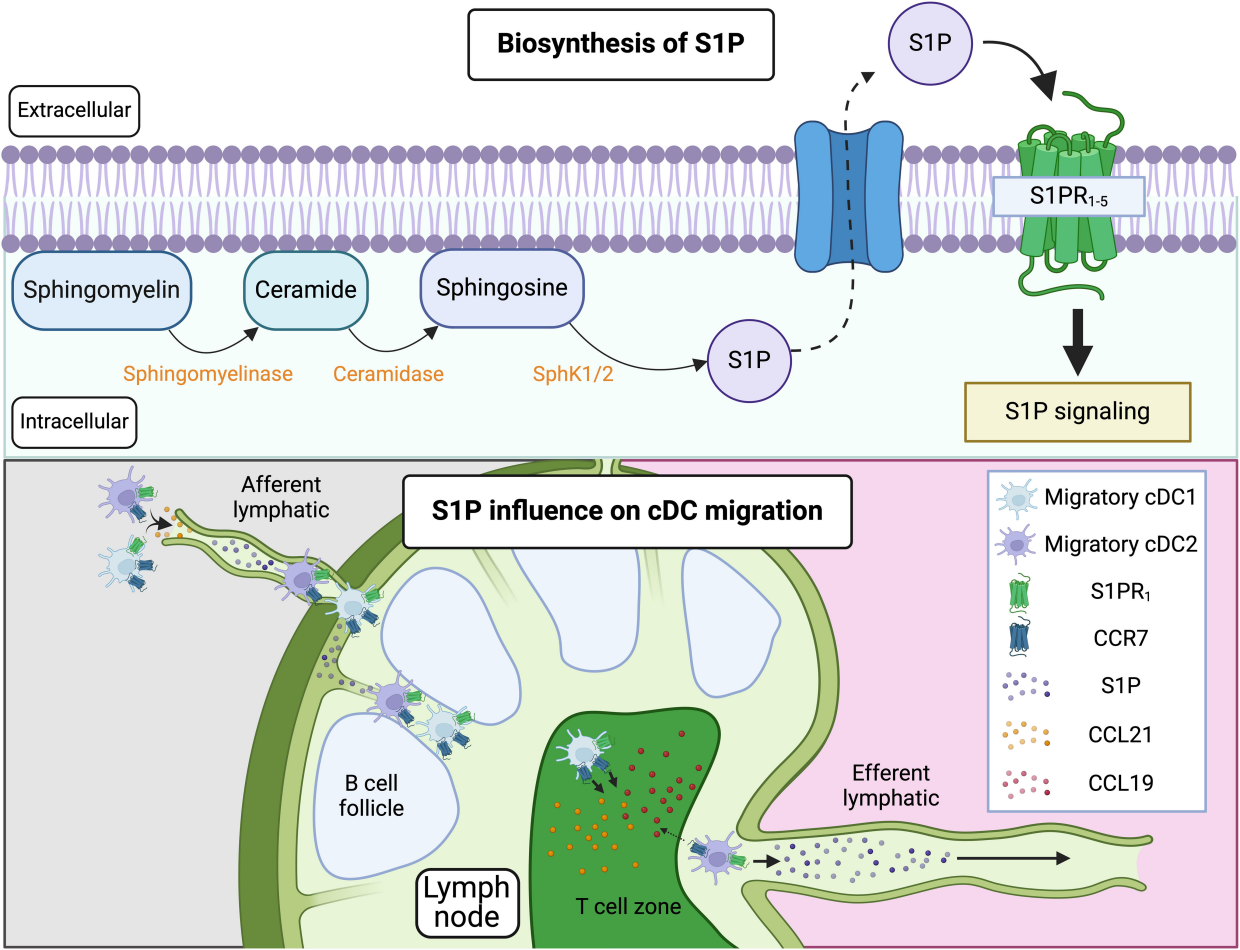
Pathway of S1P synthesis and influence on cDC migration. S1P is synthesized through the hydrolysis of sphingomyelin, a major sphingolipid in the plasma membrane, into ceramide. Ceramide is then metabolized by ceramidase into sphingosine, which is subsequently phosphorylated by SphK1/2 to produce S1P. S1P can be exported from cells via membrane transporters and acts through G-protein-coupled receptors, S1PR1-5. When S1P binds to S1PR1 on immune cells (such as T cells, B cells, and cDCs) residing in LNs, it promotes their exit from the LNs by overriding retention signals mediated by CCR7. This process involves S1P-driven migration of these cells through efferent lymphatics into the thoracic duct, ultimately reaching the bloodstream. Notably, cDC2s, but not cDC1s, migrate out of the LNs. This difference is likely due to variations in CCR7 expression, with cDC1s exhibiting higher CCR7 levels, leading to their retention in the LNs. In situations where CCR7 is not strongly upregulated on tissue cDCs, S1P/S1PR interactions may also facilitate the migration of cDCs from peripheral tissues to dLNs. Figure created with Biorender.

Sphingolipids are predominantly found in the outer leaflet of the plasma membrane, where they play a critical role in maintaining membrane structure and function. They interact with cholesterol to form lipid rafts, specialized microdomains that regulate membrane fluidity, protein localization, and signal transduction (119). In addition to their structural role, sphingolipids serve as precursors for bioactive molecules such as S1P (120). S1P is formed by the hydrolysis of sphingomyelin, a major sphingolipid in the plasma membrane, by sphingomyelinase to produce ceramide. Ceramide is then further metabolized by ceramidase, which breaks it down into sphingosine. Finally, sphingosine is phosphorylated by sphingosine kinase (SphK1 or SphK2) to generate S1P (56). S1P can be exported from cells via membrane transporters and acts as a ligand for the G-protein-coupled S1P receptors (S1PR1-5) (**Figure 3**). As such, it serves as a potent signaling molecule involved in regulating the vascular system, including vascular stability, permeability, and angiogenesis (120). Additionally, S1P regulates immune cell trafficking, particularly of T cells, B cells, and cDCs.

In the LN, S1P is available within or nearby cortical sinusoids and medullary sinuses, which are channels through which lymph flows before exiting the LN via efferent lymphatics. In T cells, S1P binding to the S1PR1 promotes LN egress by overcoming retention signals mediated by CCR7 (121–123). CCR7 ligands, such as CCL19 and CCL21, are abundantly present in T zone stromal cells and promote the motility and retention of T cells in the T cell zone. In contrast, S1PR1 guides S1PR1-expressing cells into the efferent lymphatic vessels for egress. T cells that have divided multiple times upregulate S1PR1 and downregulate CCR7, switching to a state favoring egress over retention. This same mechanism that controls the egress of lymphocytes from LNs also regulate the egress of migratory cDCs, which then traffic from the lymph via the thoracic duct to the blood and subsequently enter the spleen (124) (**Figure 3**). Importantly, only cDC2s, and not cDC1s, migrate out of the LN (124). This difference could be explained by variations in CCR7 expression between these two subsets. CCR7 counteracts S1P-mediated exit from the LN, and cDC1s characteristically express higher levels of CCR7 relative to cDC2s. Thus, cDC1s could be predominantly retained in the LN, whereas the lower expression of CCR7 in cDC2s would allow their exit.

In addition to controlling egress from the LN, some studies have shown that blocking S1P/S1PR after FTY720 treatment, which causes downmodulation and functional inhibition of S1PRs, prevents the migration of cDCs from tissues such as the skin (125–128) and the lung (129) to dLNs. However, other studies have not observed this effect and found that S1P/S1PR interactions are not required for tissue-to-LN migration (124). Importantly, studies showing the necessity of S1P/S1PR for proper cDC migration were conducted under immunization or exposure to allergens, whereas studies that did not require S1P/S1PR were conducted during infections such as influenza virus infection, where lung cDCs trafficked normally into the dLN in the absence of S1P/S1PR interactions. One possible explanation for this discrepancy is that in the highly inflammatory environment induced by influenza infection, sustained CCR7/CCL21-mediated chemotaxis is sufficient for guiding cDCs out of the lung tissues. In this scenario, potent CCR7 upregulation by cDCs after infection may not require S1P/S1PR interactions. In contrast, in other scenarios where CCR7 is less potently induced, S1P/S1PR interactions assists in the proper egress of DCs from the tissue. Alternatively, it is possible that S1P/S1PR co-opt to fully modulate CCR7 expression during specific inflammatory conditions (130). In this sense, S1PRs upregulation in DCs is potently induced by TNF-α+PGE2 (127), which are otherwise also required for upregulation of CCR7 (59), suggesting that these pathways may promote CCR7 expression in an interdependent manner. Further research is needed to understand where S1P is expressed in the tissue and the mechanism by which it enhances migration from tissue to dLN in cDCs, and in which specific scenarios it is required versus not required. Additionally, DCs express mRNA for all five known S1PRs (125, 129–131). However, activated pro-migratory DCs strongly upregulate S1PR1 as well as S1PR3, with the S1PR1 signal predominating, suggesting that, as in lymphocytes, S1PR1 may be the dominant receptor mediating migratory properties in cDCs (125–127, 130).

## The role of oxysterols in regulating cDC migration

**Figure 4:**
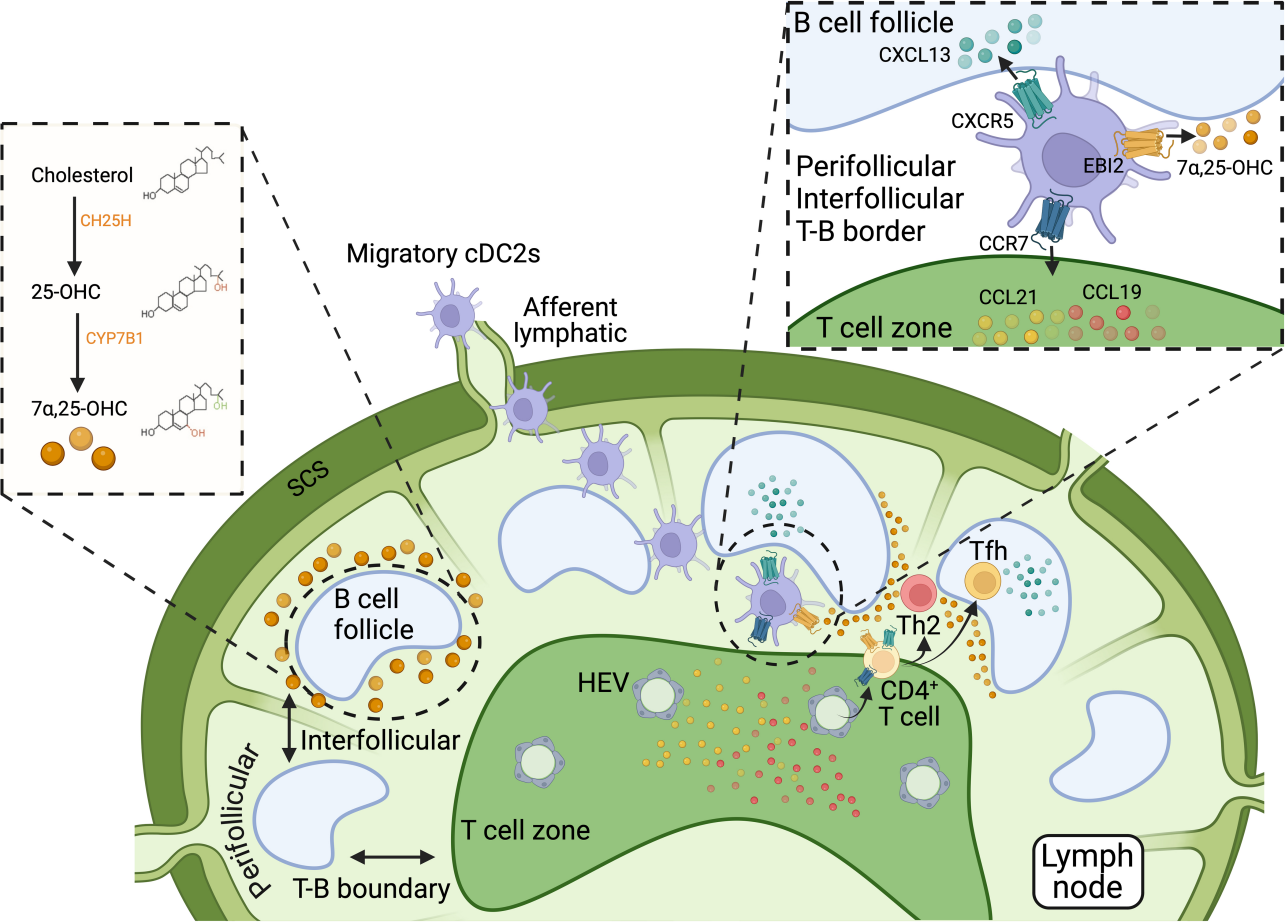
Biosynthesis and role of 7α,25-OHC in cDC localization within secondary lymphoid organs. 7α,25-OHC, an oxysterol derived from cholesterol, is crucial for the precise positioning of immune cells within secondary lymphoid tissues, such as LNs and the spleen, by acting as a ligand for the G-protein-coupled receptor EBI2 (GPR183). 7α,25-OHC biosynthesis involves two steps: cholesterol is first converted to 25-OHC by CH25H, and then 25-OHC is hydroxylated at the 7α-position by the cytochrome P450 enzyme CYP7B1 to form 7α,25-OHC. In LNs and the spleen, 7α,25-OHC is primarily produced at the borders of B cell follicles. By binding to EBI2, 7α,25-OHC directs the migration of immune cells, including B cells, T cells, and cDC2s, to perifollicular regions surrounding B cell follicles. This positioning is regulated by counteracting the chemotactic signals of two other receptor-ligand systems: CCR7-CCL19/CCL21 and CXCR5-CXCL13. CCL19 and CCL21, the ligands for CCR7, are produced in T cell areas and guides CCR7-expressing cells to these zones, while CXCL13, the ligand for CXCR5, is produced within B cell follicles and attracts CXCR5-expressing cells into the follicles. Thus, the localization of cells to T cell areas, B cell follicles, or perifollicular regions, including the T-B boundary and interfollicular locations, depends on the coordinated actions of EBI2-, CCR7-, and CXCR5-dependent signaling pathways. Migratory cDCs, particularly cDC2s, can express all three receptors—CCR7, CXCR5, and EBI2—but their expression levels vary depending on their activation state. During type 2 inflammation, low CCR7 expression combined with upregulation of CXCR5 and EBI2 facilitates the guidance of cDC2s and CD4^+^ T cells to the T-B cell boundary and peri- and inter-follicular regions in response to the ligands for these receptors. Such localization is essential for initiating Th2 and Tfh cell responses. Thus, the coordinated actions of EBI2-, CCR7-, and CXCR5-dependent signaling pathways are crucial for the effective localization and function of cDCs and cognate T cells during the initiation of type 2 immunity. Figure created with Biorender.

Oxysterols are oxygenated derivatives of cholesterol, formed through the addition of hydroxyl, carbonyl, or epoxide groups at various positions on the cholesterol molecule. These modifications significantly alter their biological activity and function compared to their parent compound, cholesterol. Oxysterols are important signaling molecules involved in numerous physiological processes, including lipid metabolism, immune response, and inflammation (132). 7α,25-Dihydroxycholesterol (7α,25-OHC) is a specific oxysterol that plays a key role in the migration of cells, including DCs. The synthesis of 7α,25-OHC occurs in two steps involving the enzymes cholesterol 25-hydroxylase (CH25H) and the cytochrome P450 enzyme CYP7B1. This pathway typically involves the initial conversion of cholesterol to 25-hydroxycholesterol (25-OHC) by CH25H, followed by hydroxylation at the 7α-position by CYP7B1 to form 7α,25-OHC (133, 134) (**Figure 4**). 7α,25-OHC is the endogenous ligand of the G protein-coupled receptor EBI2 (also referred to as GPR183) (133, 135). This receptor is expressed on various immune cells, including B cells, T cells, and DCs. By binding to EBI2, 7α,25-OHC acts as a chemotactic agent, guiding the migration of immune cells to specific locations within tissues. For instance, in LNs, 7α,25-OHC binding to EBI2, in coordination with CCR7 binding to its ligand CCL19, directs B cells to traffic into the inflamed tissue from circulation via high endothelial venules (HEV) (136). In both LNs and the spleen, this signaling also facilitates the localization of B cells at the B-T boundary (137). This positioning enables B cells to receive early help from T cells during the initiation of antibody-dependent responses (137). Subsequently, 7α,25-OHC binding to EBI2 helps guide B cells to migrate away from the B-T border following initial activation by antagonizing CCR7-mediated migration. Specifically, EBI2-dominant migration directs B cells to re-localize to interfollicular and outer follicular regions. In these regions, B cells continue to interact with T cells and receive additional signals. These sequential steps are essential for the differentiation of B cells into plasma cells during T cell-dependent immune responses (133, 137–140). Moreover, EBI2 can form heterodimers with the chemokine receptor CXCR5. This interaction has been shown to reduce the binding affinity of CXCR5 for its ligand, CXCL13, potentially influencing the spatial organization of B cells. Specifically, it has been proposed that EBI2-CXCR5 heterodimerization restricts CXCL13-mediated B cell homing into follicles, instead promoting their retention at the periphery (141). Thus, EBI2, CXCR5, and CCR7 cooperate to influence the migration and positioning of B cells within secondary lymphoid organs during immune responses.

Similarly, the binding of 7α,25-OHC to EBI2 influences the migration and positioning of DCs within secondary lymphoid tissues, where they can initiate T cell responses. Both CD4^+^ T cells and DCs, particularly cDC2s, express EBI2 and migrate towards 7α,25-OHC (133, 142–145). EBI2-driven positioning of CD4^+^ T cells and cDC2s at the T-B cell boundary in LNs is crucial for initiating T cell responses, especially in the context of type 2 immunity. The absence of EBI2 results in a lack of encounters between CD4^+^ T cells and cDC2s at the T-B border, poor priming of Th2 responses, and inadequate control of helminth infections (145). In parallel, during type 2 immunity, cDC2s upregulate CXCR5 as they downregulate CCR7 (39). CXCR5-driven positioning of CD4^+^ T cells and cDC2s at the T-B boundaries similarly controls the initial priming of Th2 responses to intestinal helminths (39). An increasing number of studies support that positioning at the T-B boundary is a hallmark of Th2 cell response initiation (4, 39, 55, 146). The evidence indicates that this localization of T cells and cDCs is orchestrated through the cooperative actions of EBI2, CXCR5, and low-level CCR7 expression (**Figure 4**). EBI2 and CXCR5 guide these cells toward follicular regions, while the limited pulling force of low CCR7 expression prevents full migration into the T cell zones but counteracts full migration into follicular and perifollicular areas driven by CXCR5 and EBI2. Thus, this cooperation and antagonistic function of CXCR5 and EBI2 with CCR7 effectively positions T cells and cDC2s at the T-B boundary, enabling the initiation of Th2 responses. Other studies also suggest that a similar location of cDC2s and CD4^+^ T cells, guided by EBI2 and CXCR5, is essential to initiate Tfh cell responses (4, 39, 144, 147–149). Further research is needed to dissect the similar and differential signals and compartmentalization of Th2 and Tfh cell responses.

## Concluding remarks

In conclusion, this review has explored the intricate dynamics of cDC migration during type 2 immune responses, emphasizing that while CCR7 is necessary, it is not sufficient on its own and requires additional signals. While CCR7 is typically the primary and sufficient driver of cDC migration from peripheral tissues to T cell areas of dLNs during homeostasis, type 1, or type 17 responses, its expression is notably low in tissue cDCs that migrate to regional dLNs during type 2 responses. We have discussed whether this low CCR7 expression necessitates additional signaling pathways or complementary mechanisms to enhance CCR7-mediated cDC migration at specific steps during their journey from tissues to dLNs. Specifically, we have discussed bioactive lipid mediators such as cysteinyl leukotrienes that cooperate with CCR7 to favor the initial entry of DCs into the afferent lymphatics in the tissues to ensure efficient arrival at the LN. Within the LN, cholesterol-derived oxysterols, particularly 7α,25-OHC, and the chemokine receptor CXCR5 ensure appropriate positioning and interaction of DCs and T cells within the T-B boundary of the LN. Importantly, we have highlighted that reduced CCR7 expression on cDC2s arriving into the LN is not merely a hindrance but is crucial for the effective induction of Th2 responses. Low CCR7 expression permits DCs to position themselves correctly within the T-B boundary LN microenvironment, preventing their positioning in the T cell area, thereby facilitating DC-T cell interactions in an environment conducive to efficient Th2 priming. This nuanced understanding of DC migration and positioning provides valuable insights into the regulation of type 2/Th2 immune responses and underscores the complexity of immune cell trafficking and function.
